# Supplementary material for: Anti-Inflammatory Effects of Idebenone Attenuate LPS-Induced Systemic Inflammatory Diseases by Suppressing NF-κB Activation
Source: Antioxidants (Basel). 2024 Jan 25;13(2):151. doi: 10.3390/antiox13020151 (PMC10885939; doi:10.3390/antiox13020151)
Supplement: Supplementary file 1 [file antioxidants-13-00151-s001.zip › antioxidants-2823483-supplementary.pdf]

Supplementary Materials for

Anti-Inflammatory Effects of Idebenone Attenuate LPS-Induced Systemic Inflammatory Diseases by Suppressing NF-κB Activation

Yumin Choi [1,2,†], Young-Lai Cho [1,†], Sujeong Park [1,2], Minkyung Park [1,2], Keun-Seok Hong [1], Young Jun Park [1,2],
In-Ah Lee [3], Su Wol Chung [4], Heedoo Lee [5] and Seon-Jin Lee [1,2,*]

[1] Environmental Disease Research Center, Korea Research Institute of Bioscience and Biotechnology (KRIBB), Daejeon 34141, Republic of Korea; u_min98@naver.com (Y.C.); ylcho@kribb.re.kr (Y.-L.C.); sjpark01@kribb.re.kr (S.P.); mingk@kribb.re.kr (M.P.); hongs06@naver.com (K.-S.H.);
pyj71@kribb.re.kr (Y.J.P.)

[2] Department of Functional Genomics, University of Science and Technology (UST), Daejeon 34113, Republic of Korea

[3] Department of Chemistry, Kunsan National University, Gunsan 54150, Republic of Korea;
leeinah@kunsan.ac.kr

[4] Department of Biological Sciences, College of Natural Sciences, University of Ulsan, Ulsan 44610, Republic of Korea; swchung@ulsan.ac.kr

[5] Department of Biology and Chemistry, Changwon National University, Changwon 51140, Republic of Korea; leehd@changwon.ac.kr

* Correspondence: sjlee@kribb.re.kr; Tel.: +82-42-879-8293

† These authors contributed equally to this work.

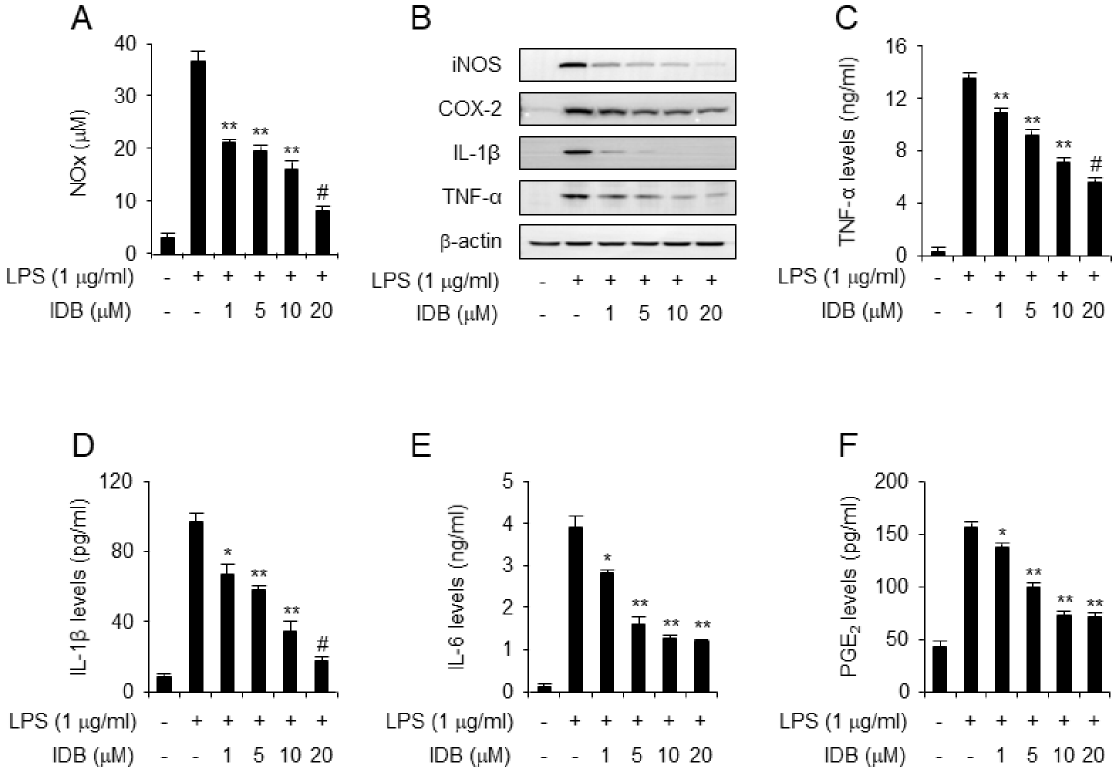

Supplementary Figure S1. Idebenone inhibits LPS–stimulated expression of proinflammatory mediators in J774A.1 cells. Idebenone inhibits LPS–induced production of NO metabolites, TNF–α, IL–1β, IL–6, and PGE_2 in J774A.1 cells. (A) NO metabolite secretion was assessed using Griess reagent. (B) Western blotting analysis was used to examined protein expression of inflammatory enzymes (iNOS, COX–2) and pro–inflammatory cytokines (IL–1β, TNF–α) in LPS–induced J774A.1 cells in the present or absence of various doses of idebenone (1, 5, 10, 20 μM), with β-actin as the loading control. Cells were pretreated with idebenone (1, 5, 10, 20 μM) for 2 h before LPS treatment (1 μg/mL). Cells were harvested after 18 h. The secretion of (C) TNF–α, (D) IL–1β, (E) IL–6, and (F) PGE_2 by LPS–stimulated J774A.1 cells treated with or without idebenone was assessed using ELISA. Graphs indicate the mean of three independent experiments. Statistical analysis was performed using paired two–tailed Student's *t*-test. *$p < 0.05$, , ** $p < 0.01$, and #$p < 0.001$.
